# Histone H3 Methyltransferase Suv39h1 Prevents Myogenic Terminal Differentiation by Repressing MEF2 Activity in Muscle Cells

**DOI:** 10.3390/ijms17121908

**Published:** 2016-11-28

**Authors:** Wei Jin, Yangyang Shang, Jian Peng, Siwen Jiang

**Affiliations:** 1 Key Laboratory of Pig Genetics and Breeding of Ministry of Agriculture & Key Laboratory of Agricultural Animal Genetics, Breeding and Reproduction of Ministry of Education, Huazhong Agricultural University, Wuhan 430070, China; rhettbutler@126.com (W.J.); guzhuyunyan@sina.com (Y.S.); 2 Department of Animal Nutrition and Feed Science, College of Animal Science and Technology, Huazhong Agricultural University, Wuhan 430070, China; pengjian@mail.hzau.edu.cn; 3 Key Projects in the Cooperative Innovation Center for Sustainable Pig Production of Wuhan, Wuhan 430070, China

**Keywords:** epigenetic modification, histone methylation, Suv39h1, protein interaction, MEF2, myoblasts differentiation

## Abstract

The myogenic regulatory factors (MRFs) and myocyte enhancer factor 2 (MEF2) transcription factors have been extensively studied as key transcription factors that regulate myogenic gene expression. However, few reports on the molecular mechanism that modulates chromatin remodeling during skeletal muscle differentiation are available. We reported here that the expression of the H3-K9 methyltransferase Suv39h1 was decreased during myoblast differentiation. Ectopic expression of Suv39h1 could inhibit myoblast differentiation, increasing H3-K9 methylation levels, whereas knockdown of Suv39h1 stimulated myoblast differentiation. Furthermore, Suv39h1 interacted with MEF2C directly and inhibited MEF2 transcription activity in a dose-dependent manner. Together, our studies revealed a molecular mechanism wherein Suv39h1 modulated myogenic gene expression and activation during skeletal muscle differentiation.

## 1. Introduction

Skeletal muscle differentiation is a rigorous development program. Genetic regulation in skeletal muscle differentiation primarily occurs at the transcription level [1]. Two key transcription factor families: myogenic regulatory factors (MRFs) and myocyte enhancer factor 2 (MEF2), are involved in this program and are required to activate downstream myogenic gene expression during muscle differentiation [2]. The MRF family includes Myf5, Mrf4, MyoD and myogenin. As master regulators of skeletal muscle differentiation, the MRF family functions through myoblast determination (Myf5 and MyoD) and differentiation (myogenin and Mrf4) [3,4,5]. In vertebrates, the MEF2 family has four members: MEF2A, MEF2B, MEF2C, and MEF2D [6]. As a transcription cofactor without myogenic activity, MEF2 acts with MRFs, to activate and sustain the myogenic differentiation program [7]. Moreover, MEF2 could perpetuate the differentiation program by regulating myogenic basic helix-loop-helix (bHLH) gene expression [8]. Histone covalent modification is an important epigenetic regulation mechanism that has essential function in chromatin structure regulation and gene expression. Histone methylation is one of these covalent modifications [9,10]. Histone deacetylases interact with MEF2 and repress myoblast differentiation [11,12]. Similar to histone deacetylation regulation, histone methylation also plays an important role in gene expression silencing. Histone H3 lysine 9 (H3-K9) methylation is the most highly studied, and it is generally associated with gene repression, X-chromosome inactivation, and heterochromatin remodeling [13]. As mammalian H3-K9-specific methyltransferase, Suv39h1 catalyzes di- and tri-methylation of H3-K9 and interacts with heterochromatin protein 1 (HP1) to ensure transcription silencing [14,15,16]. Suv39h1 is associated with MyoD and suppresses MyoD-dependent myogenic gene activity to inhibit myoblast differentiation [1].

The *Mef2c* gene is specifically expressed in muscle tissues [17]. MEF2C could regulate expression of itself and *myogenin* during myogenesis [8]. Myogenin associates with MEF2D to recruit histone acetylases, which alters the chromatin structure of late myogenic genes to promote myogenic differentiation [18], and myogenin drives high expression of myogenic genes in this loose chromatin structure [19]. In undifferentiated myoblasts, H3-K9 surrounding the MEF2 binding site at the *myogenin* gene regulatory region is highly methylated [12]. Suv39h1 is known to repress transcription and play a role in regulating myoblasts differentiation [1,20]. It is suggested that another histone modification mediates the MEF2-myogenin interaction. Therefore, we hypothesized that Suv39h1 and the associated methylation of H3-K9 suppressed MEF2-mediated myogenic differentiation by inhibiting MEF2-dependent target gene transcription.

## 2. Results

### 2.1. Suv39h1 Was Differentially Expressed during Myoblasts Differentiation

**Figure 1:**
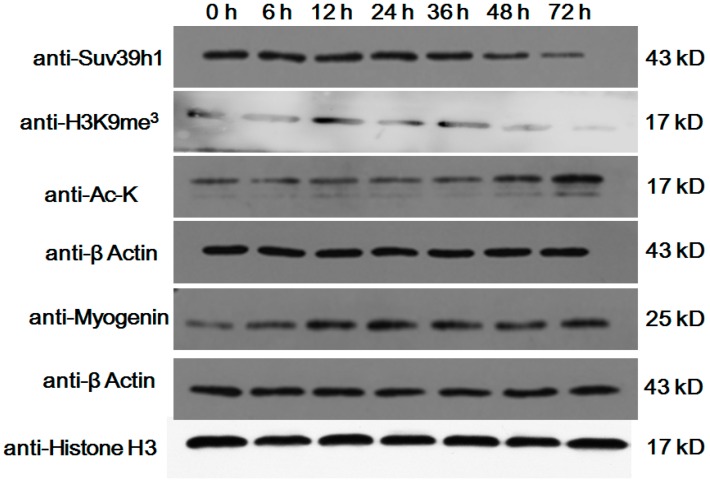
Expression of Suv39h1 during myoblast differentiation. C2C12 cells were cultured in differentiation medium for 0, 6, 12, 24, 36, 48 and 72 h. Western blot analyses were performed with cell extracts using antibodies that recognized the indicated proteins. β-actin and histone H3 were used as a loading control.

We examined the potential change in Suv39h1 expression during C2C12 cell differentiation. As shown in Figure 1, the expression of Suv39h1 protein decreased during C2C12 cell differentiation, and occurred in parallel with decreased histone methylation levels and increased histone acetylation levels. The results revealed the differential expression of Suv39h1 during myoblasts differentiation, suggesting that it might play a role in skeletal muscle differentiation.

### 2.2. Suv39h1 Inhibited Myoblast Differentiation

**Figure 2:**
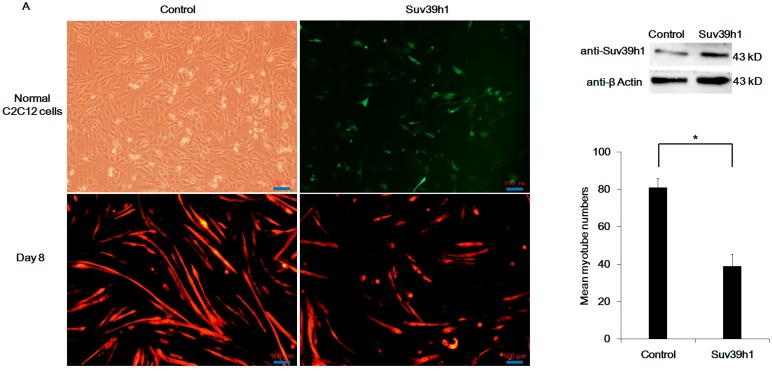

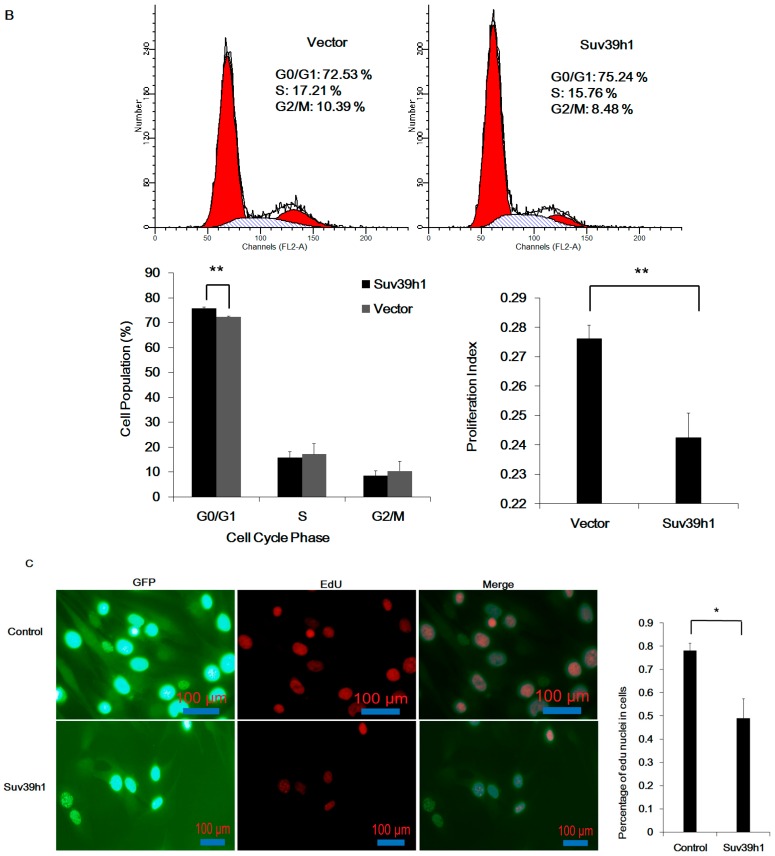
Suv39h1 affects myoblast proliferation and differentiation. (**A**) Immunofluorescence (IF) analysis of Suv39h1 in C2C12 cell differentiation. C2C12 cells were transfected with pIRES-Suv39h1 with a GFP (green fluorescent protein) expression construct or empty vector as a control and maintained in differentiation medium (2% HS (horse serum)) after transfection for 48 h. Then, the cells were stained with anti-Cy3 antibody which recognized the primary anti-MHC (myosin heavy chain) antibody (Red) after differentiation for eight days. The red Cy3-stained cells represented the level of MHC protein. Western blot analyses were performed using an anti-Suv39h1 antibody to test Suv39h1 expression. β-action was used as a loading control; (**B**) The influence of Suv39h1 overexpression on cell cycle phase of C2C12 cells. After transfection 48 h, C2C12 cells were detected by flow cytometry (FCM) and the cell cycle phase was analyzed. Then, the differences in cell populations and proliferation indices between two groups were displayed by graph. The proliferation index was calculated by (S + G_2_/M)/G_0/1_; (**C**) The influence of Suv39h1 on myoblast proliferation. C2C12 cells were transfected with pIRES-Suv39h1 or pEGFP-N1 as a control with a GFP expression and stained with EdU reagents. The GFP-positive cells (green) presented the cells transfected with pIRES-Suv39h1 or pEGFP-N1 vector. Red EdU-stained nuclei indicated proliferating, duplicating nuclei. Bars represented the mean ± SD of three experiments. The statistical significance of the differences values was analyzed by Student’s *t*-test, * *p* < 0.05; ** *p* < 0.01.

**Figure 3:**
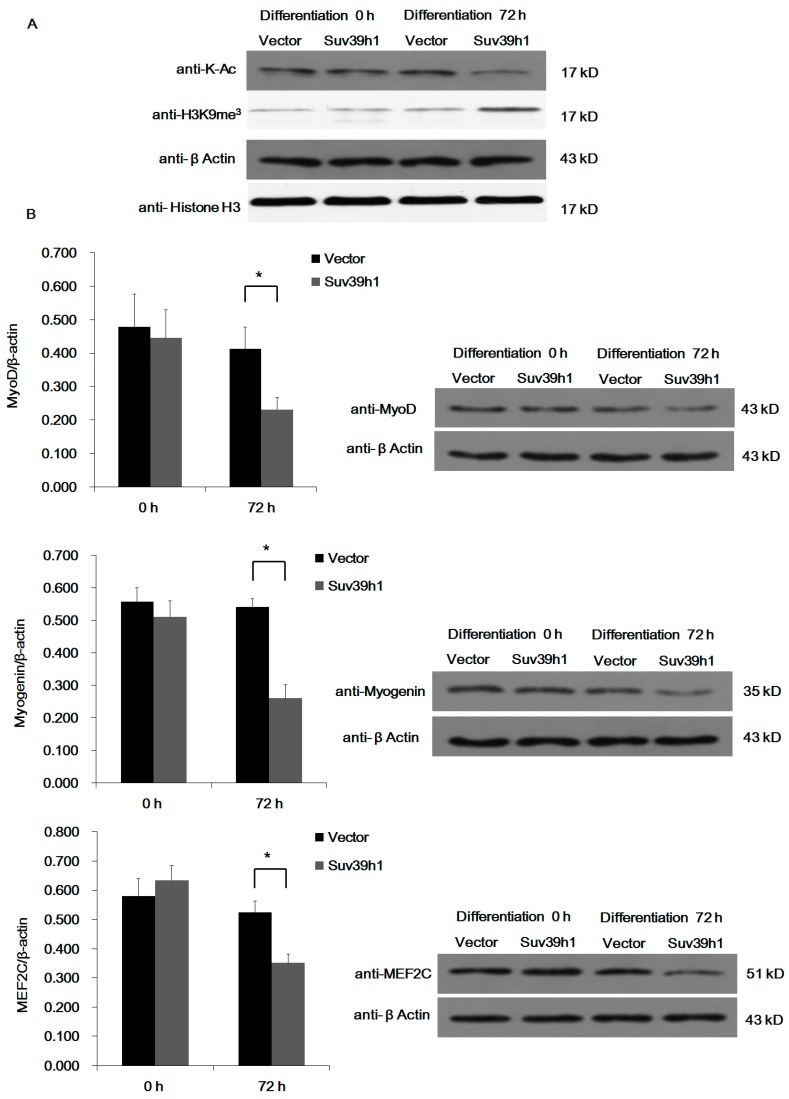
Suv39h1 inhibited skeletal muscle differentiation. C2C12 cells were transfected with pIRES-Suv39h1 or empty vector as a control. Cells were transferred to differentiation medium for the indicated time, and levels of histone modifications (**A**); and myogenic differentiation proteins (**B**) were analyzed by Western blot analysis. β-actin and histone H3 were used as a loading control. The quantification on Western blot of indicated proteins were normalized to β-actin. Bars represented the mean ± SD of three experiments. The statistical significance of the differences values was analyzed by Student’s *t*-test, * *p* < 0.05.

To test the effect of Suv39h1 on muscle differentiation, we ectopically expressed Suv39h1 in C2C12 cells. Cells were cultured in growth medium. After transfection 48 h later, the cells were cultured in differentiation medium. As shown in Figure 2A, ectopic expression of Suv39h1 appeared to block C2C12 cell differentiation, and morphological differences between Suv39h1 and the control vector transfected cells were observed. Compared with control, Suv39h1-transfected C2C12 cells exhibited reduced myotube formation. Myogenic cell proliferation and differentiation is mutually exclusive. When cell differentiation begins, myogenic genes are expressed, and myoblasts are withdrawal from the proliferation [21]. Hence, we first tested the effect of Suv39h1 on myoblast proliferation. The results showed that Suv39h1 increased amount of C2C12 cells in G_0_/G_1_-phase and the proliferation index of C2C12 cells was significantly reduced compared with control cells (Figure 2B). EdU (5-ethynyl-2′-deoxyuridine) staining assays showed that Suv39h1 might reduce new DNA synthesis in C2C12 cells (Figure 2C). Next, we analyzed the expression of early and late myogenic markers in Suv39h1-transfected cells. We also analyzed the expression of myogenic cofactor MEF2C in Suv39h1-transfected cells. As shown in Figure 3, epigenetic changes were noted in differentiated C2C12 cells. Specifically, the acetylation level decreased, whereas the methylation level increased in Suv39h1-transfected cells. The expression of myogenic differentiation markers was repressed in differentiated Suv39h1-transfected cells. Unexpectedly, the expression of early and late myogenic markers in proliferating Suv39h1-transfected cells exhibited no detectable changes. In addition, we did not detect any changes in the expression of the early myogenic marker *Myf5* in Suv39h1-transfected proliferating and differentiated cells (Figure S1).

**Figure 4:**
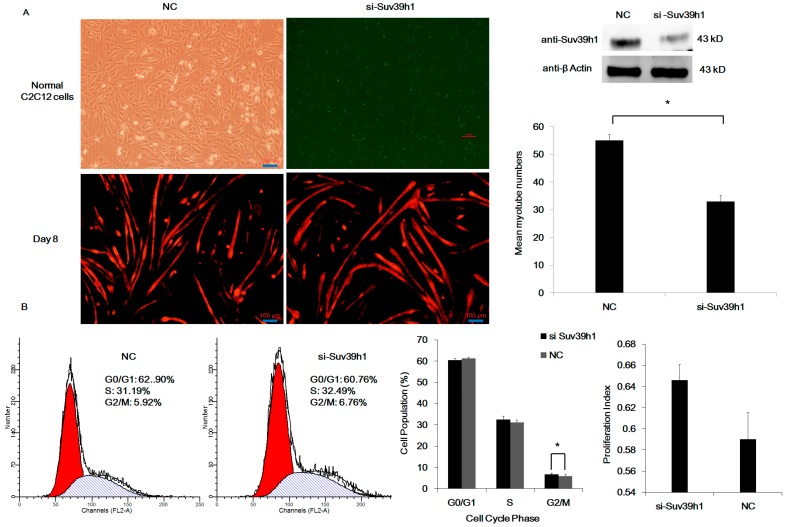

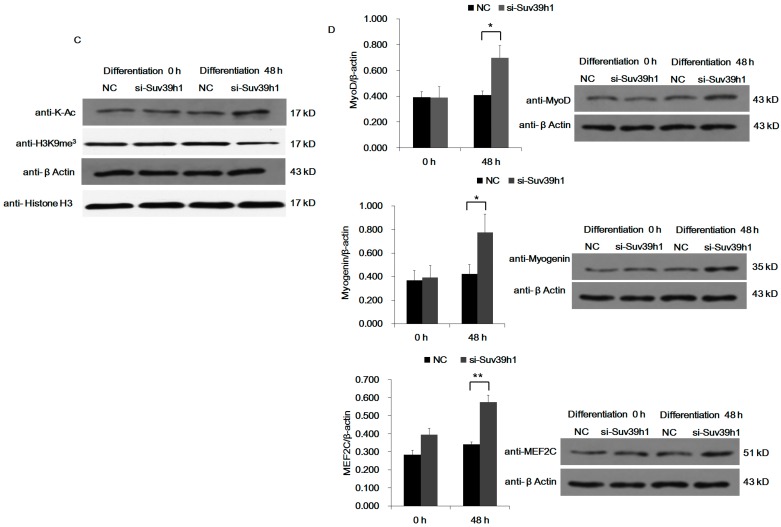
Knock-down of Suv39h1 stimulated skeletal muscle differentiation. (**A**) C2C12 cells were transfected with siRNA against Suv39h1 (si-Suv39h1) with a fluorescent label (FAM) or control siRNA (NC) as a control and maintained in differentiation medium (2% HS) after transfection for 30 h. The cells were stained with anti-Cy3 antibody which was against the primary anti-MHC antibody (Red) after differentiation for eight days. The red Cy3-stained cells presented the level of MHC protein. Western blot analyses were performed using antibody that recognized the indicated proteins; (**B**) The influence of Suv39h1 knock-down on the cell cycle phase of C2C12 cells. After transfection 48 h, C2C12 cells were detected by FCM, and the cell cycle phase was analyzed. Then, the differences in cell populations and proliferation indices between two groups were displayed by graph. C2C12 cells were transfected with siRNA against Suv39h1 or NC. Cells were transferred to differentiation medium for the indicated time, and the levels of histone modifications (**C**); and myogenic differentiation proteins (**D**) were analyzed by Western blot. β-actin and histone H3 were used as a loading control. The quantification on Western blot of indicated proteins were normalized to β-actin. Bars represent the mean ± SD of three experiments. The statistical significance of the differences values was analyzed by Student’s *t*-test, * *p* < 0.05; ** *p* < 0.01.

In addition, to confirm the aforementioned results, we knocked down endogenous Suv39h1 in C2C12 cells using siRNAs. We transfected siRNA against Suv39h1 (si-Suv39h1) or control siRNA (NC) into C2C12 cells. After eight days of differentiation, myotube numbers increased in Suv39h1 knock-down cells (Figure 4A). Suv39h1 knock-down did not affect cellular proliferation (Figure 4B). When C2C12 cells were transfected with the si-Suv39h1 or NC as a control, epigenetic changes were noted in differentiated C2C12 cells. Specifically, the acetylation level increased, whereas the methylation level decreased in Suv39h1 knock-down C2C12 cells (Figure 4C). Western blot analyses demonstrated a significant increase in MyoD, myogenin and MEF2C protein expression in differentiated Suv39h1 knock-down cells, while the expression of MyoD, myogenin, and MEF2C had no detectable changes in proliferating Suv39h1 abrogation cells (Figure 4D). Together, these data implied that Suv39h1 had a role in inhibiting the terminal differentiation of myoblasts.

### 2.3. Suv39h1 Directly Interacted with MEF2C in Undifferentiated Muscle Cells

**Figure 5:**
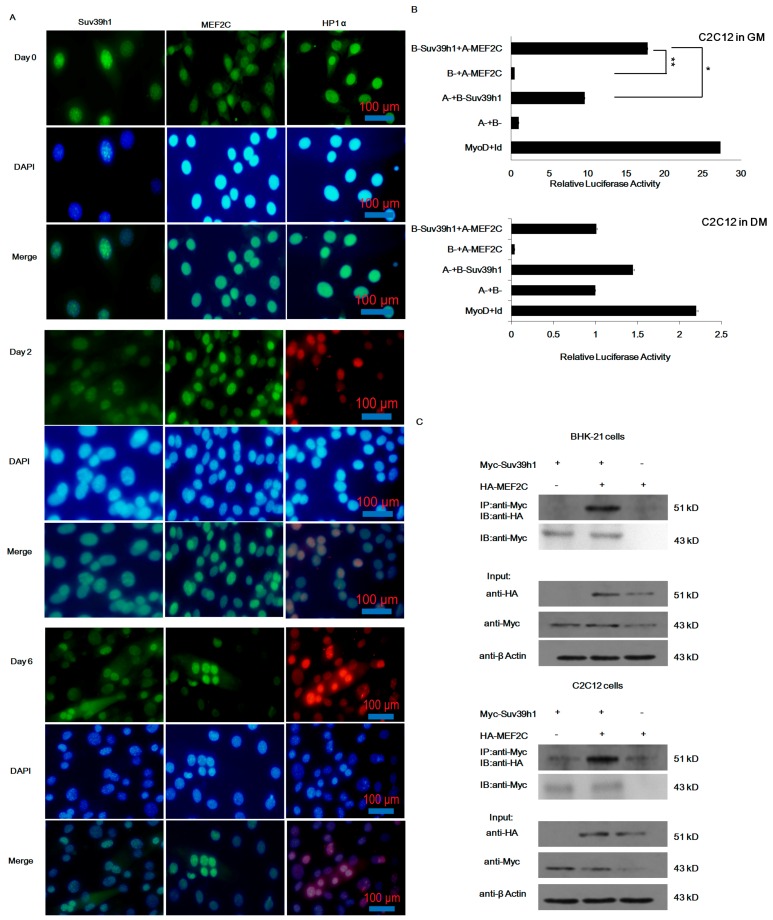
Suv39h1 and MEF2C interacted in vitro and in vivo. (**A**) Locating of Suv39h1, HP1α, and MEF2C in C2C12 cells during differentiation. The red TRITC (rhodamine) and green FITC (fluorescein isothiocyanate) antibodies were against anti-Suv39h1, anti-HP1, and anti-MEF2C antibodies as indicated, respectively. Red TRITC- or green FITC-stained cells represented the indicated proteins at indicated time; (**B**) Plasmids, including reporter plasmid pG^5luc^ were co-transfected into C2C12 cells. Then, cells were separately maintained in growth medium (GM) or differentiation medium (DM). The luciferase activity was measured 48 h after transfection. Firefly luciferase levels were normalized against Renilla luciferase levels. Bars represent the mean ± SD of three experiments. The statistical significance of the differences values was analyzed by Student’s *t*-test, * *p* < 0.05; ** *p* < 0.01; (**C**) BHK-21 and C2C12 cells were co-transfected with Myc-tagged Suv39h1 and/or HA-tagged MEF2C as indicated; Top: Suv39h1 proteins were immunoprecipitated with anti-Myc antibodies, and anti-HA antibodies were used to detect the presence of MEF2C proteins in the immunoprecipitates by Western blot analysis. Anti-Myc antibodies was used as control to detect the presence of Suv39h1 proteins; Bottom: Ten percent of cell extracts were immunoblotted to detect the presence of Suv39h1 and MEF2C proteins.

Previous studies demonstrate that MEF2 potentially recruits a histone methyltransferase to bind target gene transcriptional regions through the HP1-HDAC complex [12]. We examined whether Suv39h1 interacted with MEF2 physically and affected MEF2-mediated myogenic differentiation. Firstly, we raised antibodies against Suv39h1, MEF2C, and HP1α, and examined endogenous protein location during myoblast differentiation. In both proliferating and differentiated C2C12 cells, Suv39h1, MEF2C, and HP1α were all localized to the nucleus at indicated times (Figure 5A). Next, we applied mammalian two hybrid assays to determine the interaction between Suv39h1 and MEF2 in vivo. As shown in Figure 5B, the experiments clearly showed that Suv39h1 interacted with MEF2C in undifferentiated C2C12 cells. We further confirmed the interaction between Suv39h1 and MEF2C by co-immunoprecipitation (IP; co-IP) experiments. Then, BHK-21 and C2C12 cells were co-transfected with Suv39h1 and MEF2C expression plasmids, separately. The interaction between Suv39h1 and MEF2C was observed in both BHK-21 and C2C12 cells (Figure 5C). Together, these data suggested that Suv39h1 and MEF2 physically interacted, which further suggested that their interaction contributed to the regulation of myogenic gene expression, histone methylation modification, and myoblast differentiation. Suv39h1 might play a regulatory role through modulation of MEF2.

### 2.4. Suv39h1 Suppressed MEF2-Dependent Transcription

**Figure 6:**
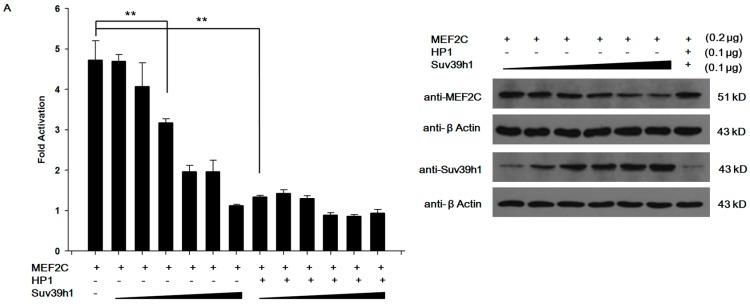

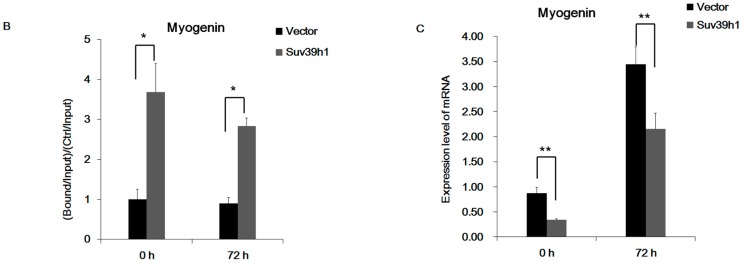
Suv39h1 inhibited MEF2 target gene transactivation. (**A**) C2C12 cells were transiently transfected with expression vectors for Suv39h1 (0.1 to 0.6 μg), MEF2C (0.2 μg), HP1α (0.1 μg), a MEF2-dependent reporter plasmid (3× MEF2-luciferase reporter plasmid; 0.1 μg), and a pRL-TK reporter (0.04 μg) to control for the differences in transfection efficiency. Firefly luciferase levels were normalized against Renilla luciferase levels. Bars represented the mean ± SD of three experiments. The statistical significance of the differences values was analyzed by Student’s *t*-test, ** *p* < 0.01. Western blot analyses were performed using antibody that recognized the indicated proteins. β-actin was used as a loading control; (**B**) C2C12 cells were transfected with pIRES-Suv39h1 or empty vector as a control. Cells were transferred to differentiation medium for the indicated time. ChIP assays were performed at differentiation 0 and 72 h on the *myogenin* promoter. Increased H3-K9me^2/3^ was apparent in Suv39h1 cells at both stages. DNA isolated from immunoprecipitated material was amplified by qRT-PCR. Total chromatin was used as the input. The results were normalized against input and *GAPDH*. Bars represented the mean ± SD of three experiments. The statistical significance of the differences values was analyzed by the Student’s *t*-test, * *p* < 0.05; (**C**) C2C12 cells were transfected with pIRES-Suv39h1 or empty vector as a control. Cells were transferred to differentiation medium for the indicated time, and expression of *myogenin* was analyzed by qRT–PCR. The results were normalized against *β-actin*. Bars represented the mean ± SD of three experiments. The statistical significance of the differences values was analyzed by Student’s *t*-test, ** *p* < 0.01.

Next, we tested whether Suv39h1 could affect MEF2-dependent transactivation. As reported, as a histone methyltransferase, Suv39h1 catalyzes H3-K9me^2^ and H3-K9me^3^, which associate with HP1 to influence gene expression [22,23]. Then, we co-transfected C2C12 cells with a luciferase reporter under three repeats of a consensus MEF2 binding site and a MEF2C expression vector along with (or without) different amounts of Suv39h1 and HP1α expression vectors. Co-transfection of Suv39h1 had an obvious effect on the expression of a MEF2C-activated luciferase reporter gene in a dose-dependent manner. The Suv39h1 inhibition effect was increased by a small amount of HP1α (Figure 6A). These data indicate that Suv39h1 potentially has the ability to inhibit MEF2-dependent target gene transactivation. Western blot experiments were performed to confirm the aforementioned observations. As shown in Figure 6A right, the large amount of Suv39h1 could reduce the expression of MEF2C. Whereas, the addition of HP1 with a small amount (0.1 μg) of Suv39h1 did not reduce the expression of MEF2C, suggesting that HP1 could be capable of repressing the MEF2C target gene. The results showed that Suv39h1 inhibits MEF2-dependent target gene transactivation via suppressing the expression of MEF2C and HP1α enhances the inhibitory action of Suv39h1 to MEF2-dependent target gene in an independent regulation manner (Figure 6A). Previous studies showed that the di- and tri-methylation of H3-K9 marks are enriched at the transcriptional region of silenced genes [24]. The expression of *myogenin* is induced during myoblast differentiation, and the transcription region of the *myogenin* gene is regulated by MEF2 [25]. Next, we performed additional independent experiments to assess whether di-/tri-methylation of H3-K9 enriched at the transcription of the *myogenin* gene was catalyzed by Suv39h1. We transfected C2C12 cells with an expression plasmid encoding Suv39h1. The results from chromatin IP (ChIP) assays showed that Suv39h1 induced H3-K9 methylation at the regulatory region of *myogenin* gene in undifferentiated and differentiated C2C12 cells (Figure 6B). Furthermore, qRT-PCR analysis showed that the expression of *myogenin* was repressed in Suv39h1-transfected proliferative and differentiated cells (Figure 6C). Taken together, we postulated that MEF2 binds to muscle differentiation genes at their regulatory regions (such as *myogenin*) to activate gene expression, whereas this muscle differentiation process was inhibited by Suv39h1.

### 2.5. HP1α Suppressed MEF2C Transcription

**Figure 7:**
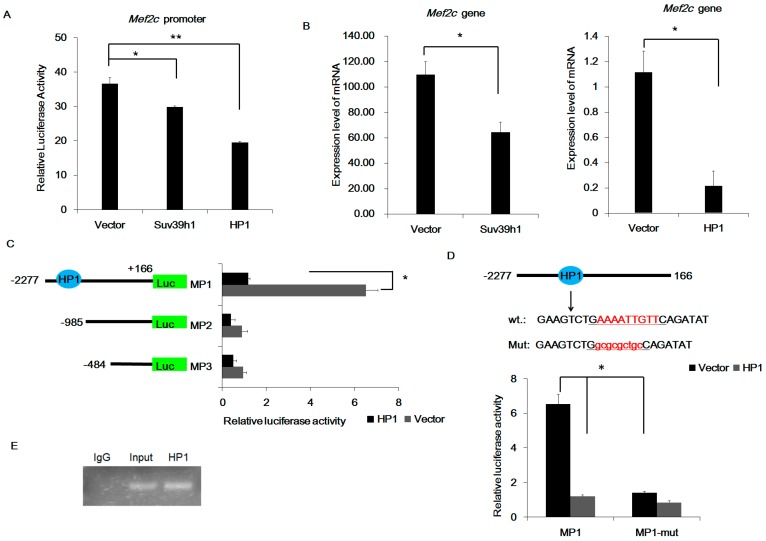
Inhibition of MEF2 transcriptional activity by Suv39h1 and HP1α. (**A**) C2C12 cells were transiently co-transfected with expression vectors for Suv39h1 or HP1α and *Mef2c* promoter vector. Firefly luciferase levels were normalized against Renilla luciferase levels. Bars represent the mean ± SD of three experiments. The statistical significance of the differences values was analyzed by Student’s *t*-test, * *p* < 0.05; ** *p* < 0.01; (**B**) C2C12 cells were transfected with expression vectors for Suv39h1 or HP1α, and the expression of *Mef2c* was analyzed by qRT–PCR. The results were normalized against *β-actin*. Bars represent the mean ± SD of three experiments. The statistical significance of the differences values was analyzed by Student’s *t*-test, * *p* < 0.05; (**C**) Effects of HP1α on *Mef2c* promoter activity. Promoter activities of a series of deleted constructs were determined by luciferase assay. The nucleotides were numbered from the potential transcriptional start site that was assigned as +1. Firefly luciferase levels were normalized against Renilla luciferase levels. Bars represented the mean ± SD of three experiments. The statistical significance of the differences values was analyzed by Student’s *t*-test, * *p* < 0.05; (**D**) The site-directed mutagenesis construct in the HP1α binding site of the *Mef2c* promoter was analyzed by luciferase assay in C2C12 cells. Firefly luciferase levels were normalized against Renilla luciferase levels. Bars represent the mean ± SD of three experiments. The statistical significance of the differences values was analyzed by Student’s *t*-test, * *p* < 0.05; (**E**) Binding of HP1α with the *Mef2c* promoter was analyzed by ChIP. DNA isolated from immunoprecipitated material was amplified by PCR. Total chromatin was used as the input. Normal rabbit IgG was used as a negative control.

Our results established that Suv39h1 inhibits of MEF2-dependent reporter gene via suppressing MEF2C expression (Figure 6A). In addition, Suv39h1 and MEF2C physically interact (Figure 5). We therefore investigated whether Suv39h1 was required to regulate MEF2C at the regulatory regions. We isolated a 2330 bp region containing 5′-flanking region of the pig *Mef2c* gene and detected a HP1 binding site in *Mef2c* promoter using TESS software (available online: http://www.cbil.upenn.edu/tess/). Unexpectedly, we did not detect a Suv39h1-binding site in *Mef2c* promoter. However, transcriptional activity of the *Mef2c* promoter was repressed by Suv39h1 in C2C12 cells, and overexpression of HP1α appeared to markedly repress *Mef2c* activity (Figure 7A). Consistent with *Mef2c* transcription activity, the *Mef2c* expression level was decreased in Suv39h1-transfected cells and HP1α-transfected cells (Figure 7B). Mutant and ChIP assays showed that HP1α was bound to the *Mef2c* promoter (Figure 7C–E). Our results suggested that Suv39h1 affected *Mef2c* gene expression indirectly, whereas HP1α affected *Mef2c* gene expression directly.

## 3. Discussion

In this study, we showed that Suv39h1 suppresses myogenic gene expression and differentiation. Ectopically expressed Suv39h1 decreased lysine acetylation and increased H3-K9 di- or tri-methylation in C2C12 cells. These data were further supported by the role of Suv39h1 in myoblasts differentiation in in vivo experiments. These results provided insights into the regulatory mechanism of how histone methyltransferases worked with myogenic transcription factors to influence myogenic gene expression and myogenic differentiation.

In this study, we investigated the effect of Suv39h1 on the muscle differentiation. We found opposite results between ours and Ait-Si-Ali and co-workers [20]. In their studies, Suv39h-depleted C2C12 cells showed a low differentiation level with reduced expression of myogenin and MHC. Ait-Si-Ali and co-workers believe that Suv39h is capable of silencing S-phase genes (cyclin D1 and A2) under low serum condition. Suv39h-depleted C2C12 cells were unable to silence S-phase genes, leading to a poor differentiation capacity. However, their data showed that “differentiating cells treated with Suv39 siRNA expressed little cyclin D1 and A2 under low serum condition”. There was a possibility that Suv39h1 up-regulated expression of cyclin D1 and A2 under low serum condition. In general, cyclins A and D1 were down-regulated upon myogenesis. A number of studies showed that overexpression of the cyclin D1, A or E could inhibit myogenic transcription [26], and this repression could be reversed by the cyclin-dependent kinase inhibitor p21 during myogenic differentiation [26]. Low serum triggers terminal differentiation in myoblasts [20]. In our studies, the myoblasts were culture in low serum to promote cell differentiation. Therefore, our data was inconsistent with Ait-Si-Ali et al [20]. In addition, the results of down-regulated of MHC by suv39h1 was supported by Mal [1]. Mal reported that Suv39h1 repressed myogenic gene expression and Suv39h-depleted C2C12 cells were able to express myogenin and MHC. Moreover, results of flow cytometry (FCM) showed that Suv39h1 arrested C2C12 cells in the G_0_/G_1_ phase of the cell cycle. Whereas, the opposite effect was not observed upon Suv39h1 knock down. Suv39h1 siRNA arrested C2C12 cells in the G_2_/M phase of the cell cycle, and neither of these affected the S phase of the cell cycle, which was involved in either DNA synthesis or cell cycle control [20]. In addition, we did not detect an opposite result from siRNA interference experiments as we expected that the proliferation index was not significantly increased in si-Suv39h1 transfected cells compared to the control cells. Moreover, the relationship between cycle control and cycle arrest was still unclear in this paper; this was also the shortcoming of this paper. Additionally, we only detected the effect of the cell population of G_0_/G_1_ phase caused by Suv39h1, and whether Suv39h affect proliferation was needed in order to detect the expression of cell cycle regulating genes. It was more complicated to find out whether the events of cell cycle exit and cell differentiation were separable [27]. Meanwhile, how to determinate the Suv39h1 as a cycle regulator to regulate cycle genes, played a role in regulating cell differentiation. These questions needed further study. Taken together, these results revealed distinct mechanisms for Suv39h regulating myoblast differentiation. Mouse embryogenesis studies indicated that MEF2C positively regulates its own expression [28], which is consistent with the auto-regulatory activity of *Drosophila* MEF2 [29]. As a MEF2C target gene, HDAC9 may associate with MEF2C, forming a negative-feedback loop that inhibits MEF2 and HDAC9 transcription and leads to the regulation of myogenic differentiation [30]. However, it is less clear how histone methyltransferases and MEF2C work together to influence myogenic gene expression. Suv39h1 associates with HP1 through tri-methylation H3-K9 [14]. A myogenesis study reported that Suv39h1 catalyzed H3-K9 methylation around the transcription region of myogenic differentiation genes [1]. Our observation indicated that Suv39h1 physically interacted with MEF2C and suppressed MEF2 target gene transactivation in a dose-dependent manner, and HP1 coordinated Suv39h1 to inhibit MEF2 target gene transactivation, whereas Western blot showed that HP1 did not coordinate Suv39h1 to down-regulate the expression of MEF2C. The studies of Zhang and colleagues showed that HP1 associated with MEF2-interacting transcription repressor (MITR) to silence MEF2 target gene, and the inhibitory action of HP1 also took place in a dose-dependent manner [12]. Hence, these data suggested that HP1 enhances the inhibitory action of Suv39h1 to MEF2-dependent target gene in an independent regulation manner. Moreover, HP1 represses *Mef2c* transcription activity and down-regulates *Mef2c* gene expression. We hypothesize that the interactions among MEF2C, HP1 and Suv39h1 might stabilize the chromatin repressive state; however, this notion must be confirmed with additional experiments.

In summary, our findings provided insights for regarding Suv39h1 repression of myogenic gene expression and myoblast differentiation, in which Suv39h1 interacts with MEF2C to regulate skeletal muscle differentiation through sustaining H3-K9 methylation at the regulatory regions of myogenic differentiation genes.

## 4. Materials and Methods

### 4.1. Plasmids Construction

Expression plasmid pcDNA3.1-Mef2c and 3× MEF2-luciferase reporter construct, which contain three upstream tandem repeats of a MEF2 binding site sequence from the *desmin* gene, were kindly provided by Eric N. Olson.

The *Suv39h1* gene was amplified using SF1-SF3 primers (Table 1). Then, the recombinant plasmids were separately digested with *Xho* I and *EcoR* I or *Sal* I and *Not* I, and ligated into the pIRES2-EGFP (BD Biosciences Clontech, Franklin Lakes, NJ, USA), pCMV-Myc (BD Biosciences Clontech), and pBIND vectors (Promega, Madison, WI, USA), separately.

The *Mef2c* gene was amplified using MF1 primers (Table 1) designed according to the *Sus scrofa Mef2c* gene (accession number: NM001044540). Then, the recombinant plasmids were digested with *Sal* I and *Not* I, and ligated into the pCMV-HA (BD Biosciences Clontech), and pACT vectors (Promega), separately.

The *HP1α* gene was amplified using HF1-HF3 primers (Table 1). Then, the recombinant plasmids were digested with *Nhe* I and *Xho* I, and *EcoR* I and *Xho* I, or *Sal* I and *Not* I, and ligated into the pIRES2-EGFP, pCMV-HA, and pBIND vectors, respectively.

### 4.2. Cell Culture and Transfection

Cells were passed into 24-well or six-well plates and were transfected with Lipofectamine 2000 reagent (Thermo Fisher Scientific, Waltham, MA, USA) according to the manufacturer’s instructions 18 to 24 h after seeding. C2C12 myoblasts were cultured in growth medium (DMEM (Dulbecco’s modified Eagle’s medium) containing 10% foetal bovine serum) (Thermo Fisher Scientific). Cells were cultured in differentiation medium (DMEM containing 2% horse serum) (Thermo Fisher Scientific) after removing the growth medium for C2C12 myoblast differentiation. Cells were incubated at 37 °C in an atmosphere of 5% CO_2_/95% air.

### 4.3. Immunofluorescence (IF) and Co-Immunoprecipitation (Co-IP) Assays

Coverslips were prepared in advance and placed in six-well plates. C2C12 myoblasts were seeded into the plates and fixed in 4% paraformaldehyde. The cell membranes were permeabilized in 0.1% (*wt*/*vol*) Triton X-100, and the cells were blocked in PBS (phosphate buffered saline) containing 3% BSA (bovine serum albumin) and stained with antibodies in PBS containing 3% BSA. Primary antibodies against Suv39h1 (Abcam, Cambridge, Cambridgeshire, UK), MEF2C (Abcam), HP1α (Abcam), and MHC (Abcam) were diluted at 1:500. Secondary fluorochrome-conjugated antibodies (Boster, Wuhan, China) were diluted at 1:64. DAPI (diamidino-phenyl-indole) was used as the nuclear counterstain.

Cells were washed three times in PBS, scraped from 10 mm^2^ culture dishes and suspended in NP40 cell lysis buffer. Cell lysates were incubated on ice by repeated pipetting for 30 min and centrifugation in a microfuge for 10 min at 13,000× *g*, at 4 °C. For co-immunoprecipitations, antibodies (2 μg anti-Myc, MilliporeSigma, Billerica, MA, USA) were incubated with 500 μL of cell extract supernatant overnight at 4 °C. Then, this immune complex was immobilized on 20 μL protein A/G agarose (Thermo Fisher Scientific) and incubated for 2 h at 4 °C. The immunoprecipitates were washed thrice in wash buffer, eluted in 20 μL elution buffer and analyzed by Western blot.

### 4.4. Chromatin Immunoprecipitation (ChIP) Assays

ChIP assays was performed as previously described [31] using 1 μg IgG (normal rabbit or mouse, Beyotime, Shanghai, China) or antibodies against HP1α (Abcam) and di/tri-methylated H3-K9 (Cell Signaling Technology, Danvers, MA, USA). The primers used in ChIP PCR included *HP1* forward, 5′-CACCCAAGCCGGGAGAAACAGCC-3′, and reverse, 5′-GTGAATCAGACAGCCTCC-3′; *myogenin* forward [11], 5′-GAATCACATGTAATCCACTGGA-3′, and reverse, 5′-ACGCCAACTGCTGGGTGCCA-3′; *GAPDH* forward, 5′-GCTCTCTGCTCCTCCCTGTT-3′, and reverse, 5′-CAATCTCCACTTTGCCACTGC-3′.

### 4.5. RNA Interference

Double-stranded small interfering RNAs (siRNAs) targeting Suv39h1 were obtained from genepharma (Shanghai, China). C2C12 cells were transfected with negative control (NC) or Suv39h1 siRNAs according to the manufacturer’s instruction. Cells were cultured in growth medium for 30 h after transfection and then switched to differentiation medium.

### 4.6. Western Blot and Quantitative Real-Time PCR (qRT-PCR)

Protein samples were analyzed by 5%–12% acrylamide gradient SDS-PAGE (SDS, sodium dodecyl sulfate; PAGE, polyacrylamide gel electrophoresis) gel and transferred to a PVDF (polyvinylidene fluoride) membrane in transfer buffer. The membrane was blocked in 5% skim milk and incubated overnight at 4 °C with the primary antibodies. Primary antibodies against HA (Cell Signaling Technology), Myc (MilliporeSigma), Myogenin (Abcam), MyoD (Santa Cruz Biotechnology, Santa Cruz, CA, USA), MEF2C (Abcam), Suv39h1 (Abcam), H3-K9me^2/3^ (Cell Signaling Technology), and Acetyl-Lys (Ac-K) (Cell Signaling Technology) were diluted at 1:1000. The appropriate secondary antibodies (Boster) recognized the primary antibodies and the membrane was visualized by using an ECL (enhanced chemiluminescence) kit (Thermo Fisher Scientific).

Total RNA was extracted from measured samples by TRIzol reagent (Thermo Fisher Scientific). After purification, 1 μg RNA was used for reverse transcription with oligo (dt) primers. qRT-PCR was run by the LightCycler^®^480 II (Roche, Basel, Switzerland) using SYBR^®^Green Real-Time PCR Master Mix (Toyobo, Osaka, Japan). All PCRs were repeated in three experiments, and gene expression was corrected by the expression of *β-actin* using 2^−△△^*^C^*^t^ value. Student’s *t*-test was used for statistical comparisons. A *p*-value < 0.05 indicated statistical significance. Primers used in the qRT-PCR are presented in Table 2.

## 5. Conclusions

Our study helped to understand how histone methyltransferases control myogenenic gene expression during myoblast differentiation. Unexpectedly, we did not detect an opposite result from siRNA interference experiments, and our results were inconsistent with Ait-Si-Ali and co-workers. It was the shortcoming of this paper, and these questions needed further study. Next, we should investigate the molecular mechanism for the association of Suv39h1 with Rb/EF2 during myoblast differentiation under our laboratorial situation, which would happen at the G_1_/S phase transition. The present study has shown that Suv39h1 as a tumor suppressor of reducing rhabdomyosarcoma formation in zebrafish [32], which could be used for cancer therapy. Since Suv39h1 also is involved in chromatin remodeling, cell lineage commitment, proliferation and differentiation [33], and influences cell cycle genes [20]. Thus, we suggest that Suv39h1 functions as key switch by modulating chromatin conformation, and controlling cell cycle progression, cell proliferation and differentiation. Moreover, studying Suv39h1 may have therapeutic value in further.

## Figures and Tables

**Table 1:** Primers used in in the construction of expression vectors of porcine *Suv39h1*.

Primer Name	Primer Sequences (5′–3′)	Size (bp)	AT (°C)
SF1	**CCG**CTCGAGATGGCGGAAAATTTAAAAGGA	1239	68
SR1	**G**GAATTCCTAGAAGAGGTATTTGCGGCA		
SF2	**G**GAATTCATGGCGGAAAATTTAAAAGGAT		
SR2	**CC**CTGCGAGCTAGAAGAGGTATTTGCGGCA		
SF3	**GC**GTCGAC*gccgccacc*ATGGCGGAAAATTTAAAAGGAT		
SR3	**TT**GCGGCCGCCTAGAAGAGGTATTTGCGGCA		
MF1	**GC**GTCGAC*gccgccacc*ATGGGGAGAAAAAAGATT	1313	64
MR1	**TT**GCGGCCGCTCATGTTGCCCATCCTTCAG		
HF1	**CTA**GCTAGCATGGGAAAGAAAACCAA	576	68
HR1	**CCG**CTCGAGTTAGCTCTTTGCTGTTTCTT		
HF2	**G**GAATTCATGGGAAAGAAAACCAA		
HR2	**CCG**CTCGAGTTAGCTCTTTGCTGTTTCTT		
HF3	**GC**GTCGAC*gccgccacc*ATGGGAAAGAAAACCAA		
HR3	**TT**GCGGCCGCTTAGCTCTTTGCTGTTTCTT		

The *Xho* I site in SF1 and *Eco*R I site in SR1, the *Eco*R I site in SF2 and *Xho* I site in SR2, the *Sal* I site in SF3 and *Not* I site in SR3, the *Sal* I site in MF1 and *Not* I site in MR1, the *Nhe* I site in HF1 and *Xho* I site in HR1, the *EcoR* I site in HF2 and *Xho* I site in HR2, and the *Sal* I site in HF3 and *Not* I site in HR3 are underlined, respectively. The protective base pairs are presented in bold. The Kozak sequence is noted in italic.

**Table 2:** The primers for real-time PCR.

Gene	Primer Sequences (5′–3′)	Size (bp)	AT (°C)
*Suv39h1*	ACCTGTGCCGACTAGCCAAGCCACGCCACTTAACCAGGTA	136	60
*Mef2c*	TGTGCGACTGTGAGATTGTCTCGTGCGGCTCGTTGT	123	59
*β-actin*	GGCACCACACCTTCTACAATGGGGGTGTTGAAGGTCTCAAAC	133	60

## Supplementary Materials

Supplementary materials can be found at www.mdpi.com/1422-0067/17/12/1908/s1.
