# Supplementary material for: Histone H3 Methyltransferase Suv39h1 Prevents Myogenic Terminal Differentiation by Repressing MEF2 Activity in Muscle Cells
Source: Int J Mol Sci. 2016 Nov 28;17(12):1908. doi: 10.3390/ijms17121908 (PMC5187760; doi:10.3390/ijms17121908)
Supplement: Supplementary file 1 [file ijms-17-01908-s001.pdf]

Supplementary Material: Histone H3 Methyltransferase Suv39h1 Prevents Myogenic Terminal Differentiation by Repressing MEF2 Activity in Muscle Cells

Wei Jin, Yangyang Shang, Jian Peng and Siwen Jiang

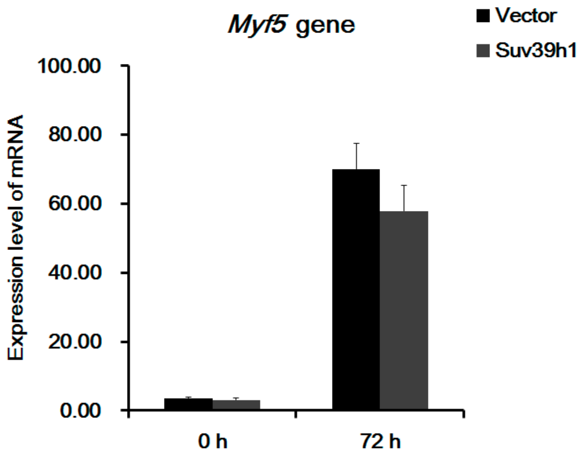

Figure S1. C2C12 cells were transfected with pIRES-Suv39h1 or empty vector as a control. Cells were transferred to differentiation medium (2% HS) for the indicated time, and expression of *Myf5* was analyzed by qRT-PCR. The results were normalized against *β-actin*. Bars represented the mean ± SD of three experiments. The statistical significance of the differences values was analyzed by Student's *t*-test.
